# Breast Reconstruction Using the Extended Latissimus Dorsi Myocutaneous Flap—A Long-term Follow-up Utilizing BREAST-Q

**DOI:** 10.1093/asjof/ojae002

**Published:** 2024-01-16

**Authors:** Alexander Wyckman, Armin Assareh, Ingrid Steinvall, Johann Zdolsek

## Abstract

**Background:**

The latissimus dorsi (LD) flap is a commonly used method for breast reconstruction after mastectomy. In this study, we present a long-term follow-up and effects of refining surgery on patient satisfaction and quality of life after breast reconstruction with the extended LD flap, using the BREAST-Q questionnaire.

**Objectives:**

The aim of this study was to investigate the patient-reported long-term results after breast reconstruction with the extended LD myocutaneous flap.

**Methods:**

A retrospective cohort study of adult patients (*n* = 101) who were operated on using the extended LD flap for breast reconstruction at the Linköping University Hospital during 1997 to 2012 was made using data retrieved from medical records. The patients were asked to complete the BREAST-Q questionnaire at 2 different postoperative time points.

**Results:**

Eighty-three patients replied to the first questionnaire, and 56 patients also replied to the second. Mean follow-up was 11.7 years. Higher age and living together with someone correlated to higher BREAST-Q results, while postoperative infection, bilateral LD flaps, smoking, and prior breast surgery had a negative impact on the results. Overall BREAST-Q results increased over time. No independent effect of refining surgery could be shown.

**Conclusions:**

Patient satisfaction after breast reconstruction with the LD flap as measured with the BREAST-Q questionnaire is high and in line with other studies. The overall satisfaction with the reconstruction method seems to increase with time, but no further increase in satisfaction after refining surgery could be established.

**Level of Evidence: 3:**

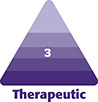

The latissimus dorsi (LD) myocutaneous flap was introduced by Tansini as early as 1906^1-3^ and may have been the first autologous method of breast reconstruction. Today, the deep inferior epigastric perforator (DIEP) flap is considered the gold standard of autologous breast reconstruction and the LD flap has become a secondary choice. One of the reasons for this is that the standard LD flap alone does not provide sufficient volume and an implant is commonly added.^4-10^ The use of an implant, however, can cause long-term complications, in particular capsular contracture, which has been seen in 20% to 40% of patients.^5,6,11^ Other implant-related complications include implant migration, infection, or even implant extrusion.^12-16^

In order to avoid these complications and to increase flap volume, several authors have proposed modifications of the flap design leading to the “extended LD flap.” The first attempts were made by including lumbar fat extensions and were described by Hokin in 1983.^17^ This technique was then modified by McCraw and Papp, introducing the “fleur-de-lis” skin paddle design. This modification increases the volume and adaptability of the flap.^18-20^ In recent years, fat grafting of the flap has been added commonly to increase volume and improve shape, allowing the reconstruction to remain fully autologous.^21^

When compared with DIEP flaps, potential advantages of the extended LD flap include shorter operative times, shorter postoperative recovery, and lower rates of flap failure. The extended LD flap can also be useful as a salvage procedure after failed autologous or prosthetic breast reconstruction.

Breast reconstruction after mastectomy plays an important role in the interdisciplinary care of the patient with breast cancer. As breast reconstructions have become more common, the demand for information regarding patient-related outcomes has increased. To better measure healthcare outcomes, patient-reported outcome measures have become an essential part of quality care.^22^ The BREAST-Q is a questionnaire that specifically measures body image and quality of life in breast reconstruction patients.^23-25^

Although the BREAST-Q has been widely used for the evaluation of patient satisfaction and quality of life following different types of breast reconstructive surgery, there are still few studies on the long-term outcome of the extended LD myocutaneous flap.^26^ It has not been clarified if an additional improvement in BREAST-Q scores can be observed following secondary refining surgery after the initial reconstruction.

The aim of this study was to investigate the patient-reported long-term results after breast reconstruction with the extended LD myocutaneous flap. We also wanted to investigate whether repeated BREAST-Q measurements would show the effect of any refining surgery performed between the surveys.

## METHODS

A retrospective analysis was conducted of all consecutive patients (*n* = 101) undergoing breast reconstruction using the extended LD flap at Linköping University Hospital, between February 1997 and December 2012. The inclusion criteria were females who had undergone total mastectomy followed by immediate or delayed breast reconstruction with the extended LD flap. Both unilateral and bilateral breast reconstructions were included. Patients who had received extended LD flaps for other reasons, such as reconstruction of Poland's syndrome or reconstruction of partial mastectomy defects were excluded.

Patients chosen for breast reconstruction with the extended LD flap were primarily those who wished or needed an autologous breast reconstruction but were not suitable for DIEP reconstruction due to obesity, previous abdominal surgery, medical comorbidity, or advanced age. Conversely, a thin body build, perceived dependence on LD muscle function, chronic neck pain, chronic shoulder pain, and arm lymphedema were considered relative contraindications for the use of the extended LD flap.

Demographics, preoperative, and postoperative data were collected retrospectively by reviewing medical records. Collected data included: age, height, weight, breast cancer treatment, immediate and late complications, direct or delayed reconstruction, and additional breast surgery.

The study was approved by the Regional Ethics Review Board (No. 2018\18-31).

### Surgical Technique

**Figure 1.**
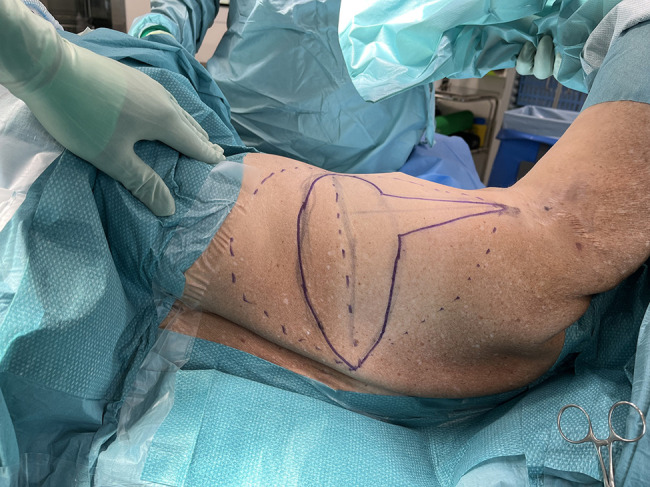
Flap design. The black dashed lines outside the skin incision lines represent the inclusion of adipose tissue below the fascia of Scarpa to increase flap volume.

The flap design was marked preoperatively with the patient upright. Attainable volume of the flap was estimated by the pinch test, and a modified fleur-de-lis skin incision pattern was drawn. The horizontal incision lines were drawn so that the postoperative horizontal scar would be concealed by the bra strap. A vertical triangle extension cranially of the skin paddle was placed over the posterior axillary fold, facilitating access to the thoracodorsal pedicle, and adding additional flap volume (Figure 1, Video).

The flap was raised, and the reconstruction was completely performed with the patient in the lateral decubitus position. The skin was incised down to the plane of Scarpa's fascia, and the dissection was continued in that plane, incorporating as much adipose tissue as possible without compromising donor-site blood supply excessively. In all patients, the humeral insertion of the muscle was preserved. The thoracodorsal nerve and its accessory branches were identified and resected to prevent involuntary postoperative muscle animation.

The completely raised flap was then rotated anteriorly and tunneled under the axillary skin to fill in the defect in the breast. The breast- and donor-site drains were removed when output was <50 mL in 24 h.

Refining surgery included liposuction and fat grafting, scar revisions, and nipple areolar complex (NAC) reconstruction, if this had not been performed earlier, and other minor adjustments.

### BREAST-Q

The BREAST-Q module used in this study was specific to breast reconstruction using the LD flap. This instrument encompasses 6 domains: (1) satisfaction with breasts, (2) satisfaction with outcomes, (3) psychosocial well-being, (4) sexual well-being, (5) satisfaction with back appearance, and (6) satisfaction with shoulder and back function. The scoring of the BREAST-Q was performed using QScore, which was developed according to the Rasch model. For domains “satisfaction with back appearance” and “satisfaction with shoulder and back function,” conversion tables were used. All domains are scored from 0 to 100, with higher scores indicating greater satisfaction or function. BREAST-Q version 1.0 was used in this study.

BREAST-Q questionnaires, and a self-addressed postage-paid return envelope, were sent at 2 different time points. The first questionnaire was sent at least 1 year after breast reconstruction surgery and the second questionnaire 7 years later. Nonresponding patients were contacted by telephone to remind them of the questionnaire, and in few patients, the questionnaires were answered by the patients during these telephone interviews.

Additional patient information collected with the questionnaire included marital status (living alone or living with somebody), level of education and employment status, current weight, additional breast surgery or other surgery in the back, shoulder or chest area during the follow-up period, prevalence of diseases or injuries causing back and/or shoulder pain, and recurrence of breast cancer in the patients with prior breast cancer.

Before initiating the study, the questionnaires were translated to Swedish in accordance with the MAPI Research Trust guidelines.

### Statistics

Descriptive data are presented as median (25th-75th centiles) unless otherwise stated. Probabilities of <0.05 were accepted as significant. The distribution was tested with the Lilliefors test for normality. The significance of differences between the groups was assessed using the Mann–Whitney *U* and the *χ*^2^ tests.

Multivariate linear regression was used to assess the effects of age, BMI, smoking, prior breast surgery, follow-up time, additional surgery, marital status, employment status, educational level, and clinical variables related to breast reconstruction, on the scores of the different BREAST-Q domains. The variables were analyzed with manual stepwise forward method.

## RESULTS

**Figure 2.**
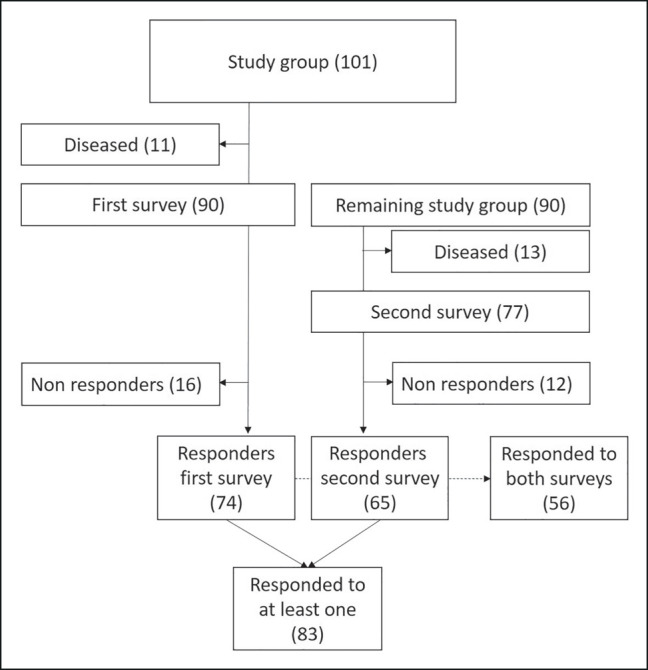
Patient selection: for the analysis of long-term outcome, the latest response on the BREAST-Q questionnaire was used among those who responded to at least 1 questionnaire; for the investigation of the effect of refining surgery, those where both BREAST-Q questionnaires had been responded to were used.

One hundred and one patients were included in the study. Eighty-three patients responded to at least 1 of the 2 the BREAST-Q surveys and 56 responded to both surveys (Figure 2).

**Figure 3.**
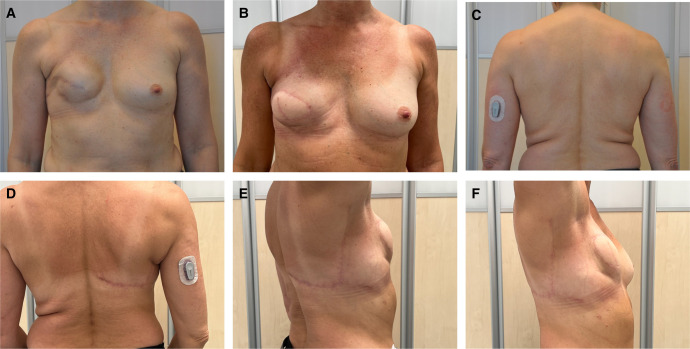
A 54-year-old female patient who underwent breast reconstruction with the extended LD flap. The patient is planned for supplemental fat grafting and nipple areola complex reconstruction. (A) Preoperative photograph, frontal view. (B) Postoperative photograph, frontal view. Eight weeks postoperatively. (C) Preoperative photograph, back view. (D) Postoperative photograph, back view. Eight weeks postoperatively. (E) Postoperative photograph, lateral view. Eight weeks postoperatively. (F) Postoperative photograph, lateral view. Eight weeks postoperatively. LD, latissimus dorsi.

Bilateral breast reconstructions were performed in 12 of the patients in the study group. Seventy-four of the reconstructions were delayed reconstructions and 9 were immediate reconstructions. Median patient age at the time of breast reconstruction was 52 years (interquartile range, 48-59 years). Median follow-up time, the time from breast reconstruction to the last survey the patient answered, was 11.7 years (interquartile range, 9.7-14.8 years). Median age at follow-up was 65 years (interquartile range, 59-72 years; Table 1). An example of a postoperative result is shown in Figure 3A-F.

**Table 1.** Description of the Patients (*n* = 83)

Follow-up time, years	11.7 (9.7-14.8)
Age, at breast reconstruction	52.0 (48.0-59.0)
Age, at survey	65.0 (59.4-72.5)
BMI, at breast reconstruction	25.4 (23.3-26.9)
BMI, at survey	25.8 (23.8-27.8)
Breast surgery prior to LD, same side	18 (21.7)
Breast implant	12 (14.5)
Breast reduction	3 (3.6)
DIEP flap	2 (2.4)
Lateral thoracodorsal flap	2 (2.4)
Breast surgery after LD	
Minor surgical refinements	46 (55.4)
NAC reconstruction	66 (79.5)
Ipsilateral breast implant	14 (16.9)
Contralateral mastopexia	8 (9.6)
Contralateral breast reduction	20 (24.1)
Contralateral breast implant	5 (6.0)
Bilateral LD reconstructions	12 (14.5)
Axillary node clearance	
Unilateral	53 (63.9)
Bilateral	3 (3.6)
Cytostatic treatment	24 (28.9)
Radiotherapy	45 (54.2)
Smoking	11 (13.3)
Postoperative wound infection	16 (19.3)
Cohabitation	60 (72.3)
Education level	
Lower secondary school	17 (20.5)
Higher secondary school/high school	28 (33.7)
University	36 (43.4)
Employed/working	30 (36.1)

Data are median (25th-75th centile) or *n* (%).

### Long-term Follow-up With the BREAST-Q

**Figure 4.**
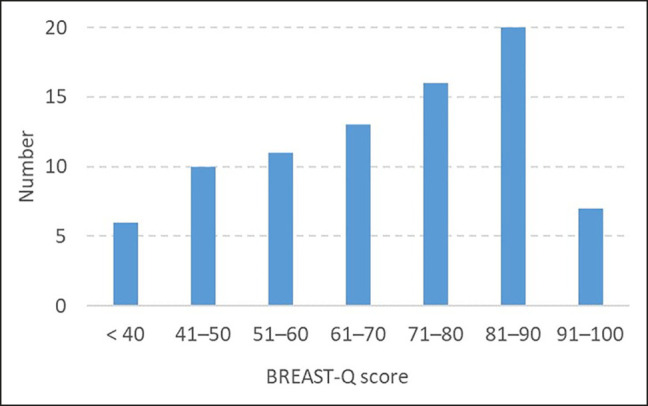
Overall BREAST-Q scores. The bars show the number of patients by score group.

BREAST-Q scores are presented in Table 2 and Figure 4. Multivariate linear regressions were performed for each BREAST-Q domain as well as for the mean score value of all domains.

**Table 2.** Long-term BREAST-Q Scoring (*n* = 83)

Satisfaction with breasts	59 (50-71)
Satisfaction with outcome	75 (51-100)
Psychosocial well-being	79 (55-100)
Sexual well-being	63 (43-90)
Satisfaction with back	78 (55-100)
Physical well-being: back and shoulder	65 (53-79)
Mean BREAST-Q score	71 (53-83)

Data are median (25th-75th centile).

### BREAST-Q Satisfaction With Breasts Domain

Cohabitation and older age (at the time of the survey) showed a positive effect on BREAST-Q scores. NAC reconstruction showed a tendency to a positive effect. Prior breast surgery before reconstruction with the extended LD flap and bilateral LD flaps showed a tendency to a negative effect on BREAST-Q satisfaction scores (Supplemental Table 1).

### BREAST-Q Satisfaction With Outcome Domain

Bilateral LD flap reconstructions showed a negative effect, whereas cohabitation and older age (at the time of the survey) had a positive effect on BREAST-Q outcome scores (Supplemental Table 2).

### BREAST-Q Psychosocial Well-being Domain

Prior breast surgery before reconstruction with the extended LD flap, bilateral LD flaps, and smoking showed a negative effect, whereas cohabitation and older age (at the time of the survey) had a positive effect on BREAST-Q psychosocial well-being scores (Supplemental Table 3).

### BREAST-Q Sexual Well-being Domain

Prior breast surgery before reconstruction with the extended LD flap, smoking, and bilateral LD reconstructions showed a negative effect, whereas older age (age at the time of the survey) had a positive effect on BREAST-Q sexual well-being scores (Supplemental Table 4).

### BREAST-Q Satisfaction With Back Appearance Domain

Postoperative wound infection showed a negative effect, whereas cohabitation and older age (at the time of the survey) had a positive effect on BREAST-Q satisfaction with back appearance scores (Supplemental Table 5).

### BREAST-Q Satisfaction With Shoulder and Back Function Domain

Prior breast surgery before reconstruction with the extended LD flap, smoking, and postoperative wound infection showed a negative effect, whereas time elapsed since reconstruction showed a tendency of a positive effect on BREAST-Q satisfaction with shoulder and back function scores (Supplemental Table 6).

### BREAST-Q Mean Score of All Domains

Prior breast surgery before reconstruction with the extended LD flap, bilateral LD flaps, smoking, and postoperative wound infection showed a negative effect, whereas living together with someone and older age (at the time of the survey) showed a positive effect on the mean BREAST-Q scores of all domains (Supplemental Table 7).

### The Effect of Refining Surgery on Repeated BREAST-Q Measurements

The group consisting of patients who answered 2 BREAST-Q surveys (*n* = 56) is presented in Table 3. This group was subdivided into 2 groups, depending on whether they had any refining surgery during the time between the 2 surveys. Refining surgery included liposuction and fat grafting, scar revision, NAC reconstruction, and other minor adjustments on the reconstructed side. The patient-group with refining procedures was younger, had shorter follow-up time, and was still employed to a higher proportion.

**Table 3.** Difference Between First and Second BREAST-Q, by Group

	All (*n* = 56)	No refining surgery (*n* = 32)	Refining surgery (*n* = 24)	*P*-value
Follow-up time, years	12 (10-15)	14 (12-15)	10 (9-12)	>.001
Age, at reconstruction	52 (48-60)	52 (49-61)	51 (47-57)	.52
Age, at survey	65 (60-73)	67 (62-76)	61 (57-71)	.02
BMI, at survey	26 (24-28)	26 (24-29)	26 (24-28)	.38
BMI change				.26
Unchanged BMI	21 (38)	11 (34)	10 (42)	
Decreased BMI	13 (23)	10 (31)	3 (13)	
Increased BMI	22 (39)	11 (34)	11 (46)	
Bilateral LD reconstructions	8 (14)	4 (13)	4 (17)	.71
Ipsilateral breast implant	9 (16)	3 (9)	6 (25)	.15
Contralateral breast reduction	16 (29)	11 (34)	5 (21)	.27
NAC reconstruction	41 (73)	24 (75)	17 (71)	.77
Cytostatic treatment	16 (29)	8 (25)	8 (33)	.49
Prior radiotherapy	29 (52)	14 (44)	15 (63)	.16
Axillary node clearance				.43
None	20 (36)	10 (31)	10 (42)	
Unilateral	33 (59)	21 (66)	12 (50)	
Bilateral	3 (5)	1 (3)	2 (8)	
Wound complications^a^				.50
None	19 (34)	9 (28)	10 (42)	
Unilateral	33 (59)	21 (66)	12 (50)	
Bilateral	4 (7)	2 (6)	2 (8)	
Smoking	7 (13)	6 (19)	1 (4)	.22
Cohabitation	46 (82)	25 (78)	21 (88)	.49
Employed/working	19 (34)	7 (22)	12 (50)	.03

Data are median (25th-75th centile) or *n* (%). Mann–Whitney *U* test. Fisher exact test, *χ*^2^ test when appropriate. ^a^Wound complications include hematoma, seroma, and infection.

**Figure 5.**
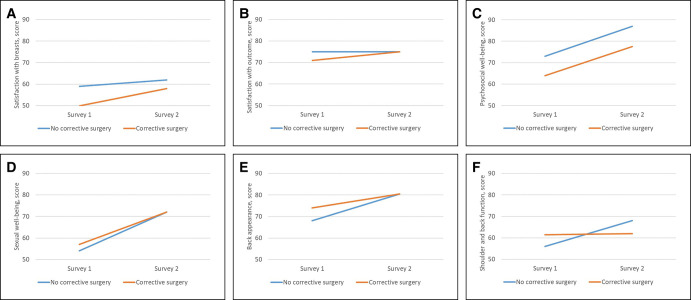
(A) Difference in BREAST-Q scoring between the first and second surveys, satisfaction with breasts domain, by group (refining surgery during the time between the 2 surveys *n* = 24, no refining surgery *n* = 32). (B) Difference in BREAST-Q scoring between the first and second surveys, satisfaction with outcome domain, by group (refining surgery during the time between the 2 surveys *n* = 24, no refining surgery *n* = 32). (C) Difference in BREAST-Q scoring between the first and second surveys, psychosocial well-being domain, by group (refining surgery during the time between the 2 surveys *n* = 24, no refining surgery *n* = 32). (D) Difference in BREAST-Q scoring between the first and second surveys, sexual well-being domain, by group (refining surgery during the time between the 2 surveys *n* = 24, no refining surgery *n* = 32) and domain. (E) Difference in BREAST-Q scoring between the first and second surveys, satisfaction with back appearance domain, by group (refining surgery during the time between the 2 surveys *n* = 24, no refining surgery *n* = 32). (F) Difference in BREAST-Q scoring between the first and second surveys, satisfaction with shoulder and back function domain, by group (refining surgery during the time between the 2 surveys *n* = 24, no refining surgery *n* = 32).

Both groups showed improvements over time in the domain psychosocial well-being and sexual well-being, whereas the domain physical well-being back and shoulder improved only in the group that did not have refining surgery (Supplemental Table 8, Figure 5). No additional effect of refining surgery between the 2 BREAST-Q surveys was found, neither by crude numbers (Supplemental Table 9) nor when adjusting for the differences in patient variables between the groups using multivariate regressions (data not shown).

**Figure 6.**
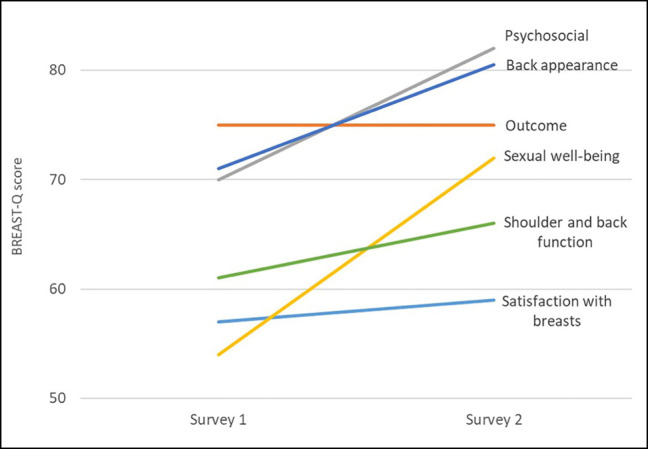
Difference in BREAST-Q scoring between first and second surveys, all patients (*n* = 56). Satisfaction with breasts *P* = .16, satisfaction with outcome *P* = .03, psychosocial well-being *P* < .001, sexual well-being *P* = .002, satisfaction with back *P* = .16, and physical well-being: back and shoulder *P* = .01.

When analyzing both groups together, significant increases in BREAST-Q scoring between the 2 surveys were observed in multiple domains. Overall BREAST-Q scores (mean values of all BREAST-Q domains were calculated for each questionnaire) showed an increased median in the second questionnaire from 63 to 72 (*P* < .001). No significant differences were observed regarding the individual domains “satisfaction with breasts” and “satisfaction with back” (Supplemental Table 8, Figure 6).

## DISCUSSION

The results of this study show that the general patient satisfaction and quality of life after breast reconstruction using the extended LD flap improves over time, according to data obtained using BREAST-Q. Although we cannot attribute this to any certain variables in our study, many factors may underlie this improvement. Time elapsed since the reconstruction and coping factors in general may allow the patient to feel more comfortable with the changes in their appearance and become more accustomed to their new situation. With increasing age and accompanying lifestyle changes, physical and aesthetic demands on the body may become less important, which may be reflected in BREAST-Q scoring also. Other studies have also shown that some negative consequences of LD reconstruction, such as limitation of shoulder mobility, may decrease over time.^27^

Living together with someone in a relationship correlated with higher BREAST-Q scores in most domains. This may be due to emotional support from the partner in a healthy relationship, which could strengthen the coping mechanisms of the patient. This could lead to a more positive view on their breast reconstruction, and a greater acceptance for pain and other discomfort related to the surgery. These patients may also have higher self-esteem and confidence, which can affect their body image and thus also BREAST-Q scoring in certain domains.

Prior breast surgery before breast reconstruction using the extended LD flap, which includes prior breast reconstruction attempts or previous cosmetic procedures (Table 1), seems to affect the BREAST-Q scores negatively. This could be explained by the fact that many of these patients had failed previous breast reconstructions, which might have a negative psychological effect. Also, scarring and altered anatomy from previous surgeries may affect the possibility and difficulty to perform breast reconstruction with the extended LD flap, and hence affect the result of the reconstruction.

Breast radiation had been received previously by 45 (54%) patients in the study group (Table 1). The effect of prior breast radiation on BREAST-Q scoring was analyzed in linear regression analyses of each individual BREAST-Q domain but yielded no significant increase or decrease in BREAST-Q scores. This is in line with findings in other studies and could reflect that prior radiotherapy is not a determining factor regarding patient satisfaction after a primarily autologous breast reconstruction.^26^

Wattoo et al. presented a similar study including 188 patients focusing on the long-term results after breast reconstruction with LD flap with a median follow-up time of 7 years. The follow-up time was longer in our study and the included patients were older, but the overall BREAST-Q scores were similar compared with our study. However, they did not utilize the LD-specific modules of the BREAST-Q.^26^ Koh et al. reported similar mean BREAST-Q scores regarding all domains, including the LD-specific satisfaction with back and physical well-being: back and shoulder, in their follow-up of 100 LD patients.^28^

Mundy et al. presented normative BREAST-Q values in a group of 1201 females without history of breast cancer or breast surgery.^29^ Compared with the normative values in this study, the scores were slightly higher in our study. This may reflect good results of the breast reconstructions performed at our department. However, our patients were also older at follow-up, which may affect the comparability between the studies.

Postoperative wound infection affects the results in the back-related domains of the BREAST-Q negatively. As postoperative infections can lead to increased scarring, which may lead to pain and discomfort as well as less pleasing results aesthetically, this association may be relevant. Postoperative complications such as wound infections have also been shown to have a negative impact on BREAST-Q scoring in other studies.^26^ Other postoperative wound problems such as hematomas and seromas did not affect BREAST-Q scoring when tested in multivariate regressions.

Interestingly, this study does not show any additional improvement in BREAST-Q scoring over time in patients who had complementary refining surgery after their breast reconstruction, between the 2 BREAST-Q surveys. The patients who had refining surgery were younger, which may reflect higher expectations and demands regarding the results of surgery. Younger patients also had a lower BMI at the time of breast reconstruction, which may be negative in the setting of the extended LD, where it is desirable to have sufficient adipose tissue to raise a flap of sufficient volume. However, when adjusting for this in multivariate regressions, there still was no significant increase in the refining surgery group. It could also be speculated that additional surgery was offered and attempted in patients who were subjectively less satisfied in general due to personality traits or other factors and that this general dissatisfaction was not altered by additional surgical interventions.

In the study by Wattoo et al, it was also found that multiple operations did not increase BREAST-Q scoring; however, they only administered the BREAST-Q questionnaire once postoperatively.^26^

### Limitations

This is a single center study with data gathering done partly retrospectively and the study group is relatively small, which may affect the generalizability of the results, and also affect the ability to find significant effects of refining surgery during the time between the 2 surveys. The study group was also heterogeneous regarding factors such as prior breast surgery before breast reconstruction. We used multivariate regression analyses in an attempt to adjust for such differences. No preoperative BREAST-Q questionnaires were administered; however, this would have been difficult due to natural circumstances regarding breast cancer diagnosis and treatment.

## CONCLUSIONS

This study shows that individual BREAST-Q results many years after breast reconstruction with the extended LD flap are good, and a gradual improvement can be seen over time. No clear effect of refining surgery on BREAST-Q scoring was found; however, more studies on this topic are needed with larger study groups and a more strictly controlled study protocol.

## Supplementary Material

ojae002_Supplementary_Data
