# Supplementary material for: Breast Reconstruction Using the Extended Latissimus Dorsi Myocutaneous Flap—A Long-term Follow-up Utilizing BREAST-Q
Source: Aesthet Surg J Open Forum. 2024 Jan 16;6:ojae002. doi: 10.1093/asjof/ojae002 (PMC11181944; doi:10.1093/asjof/ojae002)
Supplement: ojae002_Supplementary_Data [file ojae002_Supplementary_Data.zip › Supplemental Table S1-S9.docx]

| Supplemental Table S1 – Satisfaction with breasts | | | |
| --- | --- | --- | --- |
|  | Coefficient | p value | 95% CI |
| Prior breast surgery before LD | -7.3 | 0.08 | -15.3 – 0.8 |
| Age, at survey | 0.5 | 0.005 | 0.2 – 0.9 |
| NAC reconstruction | 8.3 | 0.09 | -1.2 – 17.8 |
| Cohabitation | 16.7 | <0.001 | 7.9 – 25.5 |
| Bilateral LD | -8.0 | 0.12 | -18.3 – 2.2 |
| Constant | 7.3 | 0.59 | -19.9 – 34.6 |
| Multivariable linear regression, adjusted R^2^ 0.30, p<0.001, n=81 | | | |

| Supplemental Table S2 – Satisfaction with outcome | | | |
| --- | --- | --- | --- |
|  | Coefficient | p value | 95% CI |
| Prior breast surgery before LD | -7.9 | 0.11 | -17.8 – 1.9 |
| Age, at survey | 0.8 | <0.001 | 0.4 – 1.3 |
| Cohabitation | 22.3 | <0.001 | 12.2 – 32.4 |
| Bilateral LD | -13.1 | 0.04 | -25.6 – -0.7 |
| Constant | 3.6 | 0.83 | -29.1 – 36.4 |
| Multivariable linear regression, adjusted R^2^ 0.31, p<0.001, n=79 | | | |

| Supplemental Table S3 – Psychosocial well-being | | | | |  |
| --- | --- | --- | --- | --- | --- |
|  | Coefficient | p value | | 95% CI | |
| Prior breast surgery before LD | -14.3 | 0.002 | -23.1 – -5.5 | |  |
| Age, at survey | 1.1 | <0.001 | 0.7 – 1.5 | |  |
| Smoking | -12.3 | 0.04 | -24.2 – -0.3 | |  |
| Cohabitation | 16.0 | 0.001 | 6.7 – 25.3 | |  |
| Bilateral LD | -14.7 | 0.01 | -26.1 – -3.3 | |  |
| Constant | -2.8 | 0.85 | -32.4 – 26.8 | |  |
| Multivariable linear regression, adjusted R^2^ 0.43, p<0.001, n=80 | | | | |  |

| Supplemental Table S4 – Sexual well-being | | | |
| --- | --- | --- | --- |
|  | Coefficient | p value | 95% CI |
| Prior breast surgery before LD | -19.3 | 0.004 | -32.2 – -6.5 |
| Smoking | -26.2 | 0.009 | -45.5 – -6.9 |
| Bilateral LD | -18.1 | 0.04 | -35.3 – -0.8 |
| Age, at survey | 1.1 | 0.002 | 0.4 – 1.7 |
| Constant | 7.4 | 0.73 | -34.9 – 49.6 |
| Multivariable linear regression, adjusted R^2^ 0.31, p<0.001, n=65 | | | |

| Supplemental Table S5 – Satisfaction with back appearance | | | |
| --- | --- | --- | --- |
|  | Coefficient | p value | 95% CI |
| Prior breast surgery before LD | -9.5 | 0.10 | -21.0 – 2.0 |
| Age, at survey | 0.7 | 0.009 | 0.2 – 1.3 |
| Postoperative wound infection | -20.8 | 0.002 | -33.5 – -8.1 |
| Cohabitation | 19.0 | 0.002 | 7.1 – 30.9 |
| Constant | 18.2 | 0.35 | -20.0 – 56.5 |
| Multivariable linear regression, adjusted R^2^ 0.27, p<0.001, n=80 | | | |

| Supplemental Table S6 – Satisfaction with shoulder and back function | | | |
| --- | --- | --- | --- |
|  | Coefficient | p value | 95% CI |
| Prior breast surgery before LD | -11.4 | 0.01 | -20.3 – -2.5 |
| Smoking | -12.6 | 0.047 | -25.1 – -0.2 |
| Postoperative wound infection | -18.9 | <0.001 | -29.2 – -8.7 |
| Time since LD | 0.9 | 0.057 | 0.0 – 1.7 |
| Constant | 64.3 | <0.001 | 53.0 – 75.7 |
| Multivariable linear regression, adjusted R^2^ 0.25, p<0.001, n=82 | | | |

| Supplemental Table S7 – Mean BREAST-Q scores | | | |
| --- | --- | --- | --- |
|  | Coefficient | p value | 95% CI |
| Prior breast surgery before LD | -9.9 | 0.004 | -16.5 – -3.3 |
| Age, at survey | 0.8 | <0.001 | 0.5 – 1.1 |
| Postoperative wound infection | -10.4 | 0.006 | -17.7 – -3.1 |
| Cohabitation | 16.1 | <0.001 | 9.3 – 22.8 |
| Bilateral LD | -10.9 | 0.01 | -19.3 – -2.4 |
| Smoking | -10.4 | 0.02 | -19.2 – -1.6 |
| Constant | 12.5 | 0.26 | -9.4 – 34.3 |
| Multivariable linear regression, adjusted R^2^ 0.49, p<0.001, n=81 | | | |

| Supplemental Table S8 – Inter-individual difference between BREAST-Q survey 1 and 2 | | | |
| --- | --- | --- | --- |
| No refining surgery n=32 | Survey 1 | Survey 2 | p value |
| Satisfaction with breasts | 59 (54–67) | 62 (55–70) | 0.77 |
| Satisfaction with outcome | 75 (55–86) | 75 (58–100) | 0.15 |
| Psychosocial well-being | 73 (55–89) | 87 (63–100) | 0.02 |
| Sexual well-being | 54 (37–72) | 72 (43–100) | 0.03 |
| Satisfaction with back | 68 (53–100) | 83 (64–100) | 0.15 |
| Physical well-being: back and shoulder | 56 (47–66) | 69 (53–79) | 0.003 |
| Mean all modules | 63 (53–74) | 73 (62–83) | 0.002 |
|  |  |  |  |
| Refining surgery n=24 | Survey 1 | Survey 2 | p value |
| Satisfaction with breasts | 50 (46–66) | 58 (50–65) | 0.10 |
| Satisfaction with outcome | 71 (53–75) | 75 (58–86) | 0.20 |
| Psychosocial well-being | 64 (52–78) | 78 (63–96) | 0.004 |
| Sexual well-being | 57 (39–77) | 72 (52–100) | 0.03 |
| Satisfaction with back | 74 (63–100) | 81 (58–100) | 0.60 |
| Physical well-being: back and shoulder | 62 (56–79) | 62 (54–79) | 0.81 |
| Mean all modules | 66 (55–74) | 72 (61–85) | 0.05 |
|  |  |  |  |
| All n=56 | Survey 1 | Survey 2 | p value |
| Satisfaction with breasts | 57 (50–67) | 59 (53–67) | 0.16 |
| Satisfaction with outcome | 75 (55–86) | 75 (58–100) | 0.03 |
| Psychosocial well-being | 70 (54–82) | 82 (63–100) | <0.001 |
| Sexual well-being | 54 (38–73) | 72 (47–100) | 0.002 |
| Satisfaction with back | 71 (58–100) | 83 (58–100) | 0.16 |
| Physical well-being: back and shoulder | 61 (49–71) | 67 (53–79) | 0.01 |
| Mean all modules | 63 (53–74) | 72 (62–84) | <0.001 |
| Data are presented as median (25th – 75th centile). Wilcoxon Matched Pairs Test | | | |

| Supplemental Table – S9 Differences between the groups (refining surgery or not) in first and second BREAST-Q | | | | |
| --- | --- | --- | --- | --- |
|  | All n=56 | No refining surgery n=32 | Refining surgery n=24 | p |
| The score values of Survey 1 |  |  |  |  |
| Satisfaction with breasts | 57 (50 – 67) | 59 (54 – 67) | 50 (46 – 66) | 0.06 |
| Satisfaction with outcome | 75 (55 – 86) | 75 (55 – 86) | 71 (53 – 75) | 0.56 |
| Psychosocial well-being | 70 (54 – 82) | 73 (55 – 89) | 64 (52 – 78) | 0.24 |
| Sexual well-being | 54 (38 – 73) | 54 (37 – 72) | 57 (39 – 77) | 0.96 |
| Satisfaction with back | 71 (58 – 100) | 68 (53 – 100) | 74 (63 – 100) | 0.40 |
| Physical well-being: back and shoulder | 61 (49 – 71) | 56 (47 – 66) | 62 (56 – 79) | 0.11 |
| Mean all modules | 63 (53 – 74) | 63 (53 – 74) | 66 (55 – 74) | 0.89 |
| The score values of Survey 2 |  |  |  |  |
| Satisfaction with breasts | 59 (53 – 67) | 62 (55 – 70) | 58 (50 – 65) | 0.25 |
| Satisfaction with outcome | 75 (58 – 100) | 75 (58 – 100) | 75 (58 – 86) | 0.69 |
| Psychosocial well-being | 82 (63 – 100) | 87 (63 – 100) | 78 (63 – 96) | 0.34 |
| Sexual well-being | 72 (47 – 100) | 72 (43 – 100) | 72 (52 – 100) | 0.52 |
| Satisfaction with back | 83 (58 – 100) | 83 (64 – 100) | 81 (58 – 100) | 0.83 |
| Physical well-being: back and shoulder | 67 (53 – 79) | 69 (53 – 79) | 62 (54 – 79) | 0.74 |
| Mean all modules | 72 (62 – 84) | 73 (62 – 83) | 72 (61 – 85) | 0.89 |
| Data are median (25^th^ – 75^th^ centile). Mann-Whitney U Test. | | | | |
